# PROTOCOL: Problem solving before instruction (PS‐I) to promote learning and motivation in child and adult students

**DOI:** 10.1002/cl2.1337

**Published:** 2023-07-19

**Authors:** Eduardo González‐Cabañes, Trinidad Garcia, Catherine Chase, Jose Carlos Núñez

**Affiliations:** ^1^ Department of Psychology, Psychology Faculty University of Oviedo Oviedo Asturias Spain; ^2^ Department of Human Development, Teachers College Columbia University New York New York USA

## Abstract

This is the protocol for a Campbell systematic review. The objectives are as follows: The purpose of this review is to synthesize the evidence about the efficacy of problem solving before instruction (PS‐I) to promote learning and motivation in students. Specifically, this review is designed to answer the following questions: To what degree does PS‐I affect learning and motivation, relative to alternative learning approaches? To what extent is the efficacy of PS‐I associated with the use of different design features within it, including the use of group work, contrasting cases, and metacognitive guidance in the initial problem‐solving activity, and the use of explanations that build upon students' solutions in the explicit instruction phase? To what extent is the relative efficacy of PS‐I associated with the contextual factors of activities used as control, age of students, duration of the interventions, and learning domain? What is the quality of the existent evidence to evaluate these questions in terms of number of studies included and potential biases derived from publication and methodological restrictions?

## 1 BACKGROUND

### 1.1 Description of the condition

A typical form of instruction is for teachers to explain a new concept or procedure and then ask students to apply it in a set of activities. However, some research suggests that it may be more beneficial to first give students the opportunity to problem‐solve in relation to the new contents *before* providing any explicit instruction on them. This review asks how these two approaches to instruction compare in promoting motivation and learning. For example, before explaining how to measure statistical variability, or how to solve an equation, or a psychological theory to explain our attentional experiences, would it be better if students first try to find their own solutions to these problems (e.g., Carnero‐Sierra, 2020; Fyfe, 2014; Kapur, 2012), or would it be better to start by providing them with instructions and concepts for solving the problems?

Problem‐solving activities are highly valued in education because they offer the opportunity for students to practice at their own pace (Jackson, 2021). Allowing students to take their time is an important part of the reflection processes. However, deep reflection processes are not always activated in problem‐solving activities. When students know the basic procedures to solve them, the problems often become a routine that students solve mechanically without devoting enough attention to the structural aspects (Moore, 1998). To encourage these reflection processes, it might be useful to give students the opportunity to problem‐solve before they receive any explanation about the relevant procedures (Schwartz, 2004).

These types of interventions that combine an initial phase of problem‐solving and a following phase of explicit instruction have been formulated in different specific approaches, such as the Productive Failure approach (Kapur, 2012a), the Invention approach (Schwartz, 2004), or problem‐solving before instruction (Loibl, 2014a). In this review we will generally refer to all these related approaches as problem‐solving before instruction (PS‐I).

It has been argued that PS‐I interventions can have important implications in both learning and motivation. Specifically, generating solutions in the initial problem‐solving phase can help students become more aware of their knowledge gaps (Loibl, 2014), activate and differentiate prior knowledge (Kapur, 2012), and adopt healthy motivations (Belenky, 2012). In this regard, several studies have shown that students who learned through PS‐I, in comparison to students who directly received explanations of the target concepts and procedures, reported higher interest in the content taught (Glogger‐Frey, 2015; Weaver, 2018). Also, they demonstrated higher understanding of the content and greater capacity to transfer this understanding to novel situations (Glogger‐Frey, 2017; Kapur, 2014; Schwartz, 2011; Weaver, 2018).

However, PS‐I can sometimes produce negative reactions related to learning and motivation. During the initial problem‐solving, students can feel overchallenged with the search for many possible solutions. They might spend most of the time paying attention to irrelevant aspects (Clark, 2012). Also, the uncertainty of not finding correct solutions in this task can be frustrating, and students might end up acquiring a passive role (Clark, 2012). There are some studies that have shown greater negative affect (Lamnina, 2019), lower motivation (Glogger‐Frey, 2015), and reduced learning (Fyfe, 2014; Glogger‐Frey, 2015; Newman, 2019) for students in PS‐I interventions than for students in alternative interventions.

Considering this variability of results in the literature, it is important to systematically review the evidence concerning efficacy of PS‐I, and concerning conditions that can influence this efficacy. Also, this review may have important implications for educational practice. Many instructors have negative attitudes towards the uncertainty of starting lessons with problem‐solving activities in which students can experience initial failures and negative affect (Pan, 2020), and empirical evidence of PS‐I's efficacy might help these instructors to reduce this uncertainty and take more informed decisions.

In terms of learning, it is important to evaluate to what extent PS‐I can promote the development of conceptual knowledge, which refers to the understanding of the content taught, and transfer, which refers to the capacity to apply this understanding to novel situations. Several national and international evaluations suggest that a great proportion of students learn by memorizing procedures (Mallart, 2014; OECD, 2016; Silver, 2000). For example, in the Spanish math examinations to access the university, it was noted that the majority of students passed the exams because they were able to solve problems that were similar to those seen in class. However, the majority failed to answer comprehension questions, or to correctly solve problems where they had to flexibly apply the procedures learnt (Mallart, 2014). Considering that real‐life situations generally differ from class situations, acquiring deeper forms of knowledge is of great relevance in the future autonomy of students, especially in a world that is increasingly changing because of globalization and development of new technologies (OECD, 2014).

Of no less importance is the potential efficacy of PS‐I to promote motivation for learning. Several evaluations suggest that a great proportion of students are not motivated to learn class content (Council National Research, 2003; OECD, 2018). A recent PISA evaluation with high school students from 79 countries showed that most students reported studying before or after their last class, but only 48% of these students reported interest as one of their motives (OECD, 2018). Promoting motivation for learning is of great importance, because, rather than just being a predisposition that can help learning (Chiu, 2008; Liu, 2020; Mega, 2014), it is a main factor that determines the well‐being of students during the learning process (Ryan, 2009).

Four reviews have provided interesting evidence about the comparison of PS‐I versus other educational interventions (Darabi, 2018; Jackson, 2021; Loibl, 2017; Sinha, 2021). They suggested a general efficacy of PS‐I to promote learning (Darabi, 2018), and more specifically to promote conceptual knowledge and transfer, but not procedural knowledge (Loibl, 2017; Sinha, 2021). Furthermore, the qualitative review of Loibl, 2017 and the meta‐analysis of Sinha, 2021 suggested that PS‐I was associated with higher efficacy when it was presented with guidance strategies to help students become more aware of their knowledge gaps and focus their attention on relevant features of the contents. However, these reviews are limited because they only used a small number of databases or search techniques for the identification of studies. Additionally, there are important aspects not addressed in these previous reviews, such as the evaluation of motivational outcomes, or the consideration of the types of control activities used for the comparisons.

The upcoming review aims to address these aspects and to update the studies included in these previous reviews. We will review studies in which PS‐I interventions are systematically compared with alternative interventions that provide the same contents and activities but provide explicit instruction from the beginning, and that quantify the results with conceptual knowledge tests, transfer tests, and self‐reports of motivation for learning. Yet, additional exploratory analyses might include studies either that have more general measures of learning, or in which there is not such a strict control of the equivalence of learning activities between conditions. The general goal is to provide educators and policy makers with information that can help them make decisions about introducing PS‐I in educational practice, and the various factors that can influence the efficacy of PS‐I.

### 1.2 Description of the intervention

The uniqueness of PS‐I educational interventions resides in the combination of two phases: an initial problem‐solving activity to explore a concept that students have not yet learned, and a subsequent explicit instruction phase to explain the concept. The initial problem‐solving activity consist of one or several problems that students can explore with their prior knowledge, but that require students to completely develop their own criteria to solve, because the main solution procedures are based in concepts they have not yet learned, and no content‐related guidance is given during problem‐solving. Students are not expected to find the correct solutions. However, this initial exploration is thought to prepare them to learn from subsequent explicit instruction. The second phase of explicit instruction consists of any activity in which students can read or listen to explanations of the target concepts, such as a lecture, a video, or an interactive discussion with concept explanations.

A typical approach in which PS‐I has been conducted is called Invention (Schwartz, 2004). In this approach the initial problem‐solving activity is generally formulated with invention goals, which refers to instructions to infer a general procedure, rule, or concept. Also, the data of the problem is often presented in small data sets or contrasting cases, which are examples that differ on a few key features (e.g., Section 1a of Table 1). This type of data presentation has been applied in a great variety of learning areas, including physics (Schwartz, 2011), statistics (González‐Cabañes, 2021), and educational sciences (Glogger‐Frey, 2015). The combination of invention goals and contrasting cases is meant to encourage students to discern and actively integrate relevant problem features (Schwartz, 2004; Schwartz, 2011; refer to How the intervention might work for a further description of these features).

**Table 1:** Examples of design features in problem solving before instruction (PS‐I).

PS‐I phase	PS‐I Variant 1	PS‐I Variant 2
Problem‐solving	*(1a) Contrasting cases*	*(1b) No contrasting cases*
	Which soccer player scores more consistently?	Which soccer player scores more consistently?
	The number of goals for each player across 4 seasons is shown below:	The number of goals for each player across 20 seasons is shown below:
	Player A: 9, 10, 10, 11	Player A: 14, 9, 14, 10, 15, 11, 15, 16, 12, 17, 13, 17, 13, 18, 14, 19, 14
	Player B: 5, 10, 10, 15	Player B: 13, 9, 16, 14, 10, 11, 13 14, 12, 15, 14, 17, 13, 14, 18, 14, 15
	Player C: 5, 5, 15, 15	Player C: 13, 18, 15, 10, 16, 10, 17, 19, 14, 18, 9, 10, 18, 11, 10, 18, 18
Problem‐solving	*(2a) Metacognitive Guidance*	*(2b) No Metacognitive Guidance*
	Following these instructions might help:	(No extra guidance is provided)
	‐ Rank the players by consistency	
	‐ Explain the reasons of your ranking	
	‐ How these reasons can be applied to the design of quantitative indexes to measure consistency?	
Explicit instruction	*(3a) Building on students' solutions*	*(3b) No building on students' solutions*
	Many of you calculated the sum of the deviation from one year to the next. Some used the deviations as calculated and summed them up, others took absolute values. This led to different results. What is the benefit of one solution method or the other?…	One solution that experts use is the standard deviation:
	One solution that expert use is the standard deviation:	*SD* = square root of ((*x*_*i*_ − mean)/*N*)
	*SD* = square root of ((*x_i_*− mean)/*N*)	

*Note*: Table adapted from Loibl (2017) using transformations of the PS‐I intervention in Kapur (2012) as examples.

Another typical PS‐I approach is Productive Failure (Kapur, 2012a). Studies following this approach present students with rich problems, in which the data is complex and relevant features are not highlighted (e.g., Section 1b of Table 1). The problem generally allows several possible solutions, and the ensuing explicit instruction includes explanations that build on students’ solutions, commenting on the affordances and limitations of students’ solutions in comparison to the affordances and limitations of the correct solutions (e.g., Section 3a of Table 1; refer to How the intervention might work for a further description). The Productive Failure approach has also been used in a great variety of learning areas, including physics (Kapur, 2010), statistics (Kapur, 2012), maths (Mazziotti, 2019), and biology (Song, 2018). It is emphasized in this approach that ‘failures’ to reach the correct solutions in the initial problem are not conceived as failures, but as exploration opportunities that help students activate prior knowledge, and as opportunities to comprehend relevant features and relations when these solutions are compared with the correct ones.

To give a sense of the variability across PS‐I interventions, it is also important to consider the context in which the interventions are implemented. PS‐I interventions are generally implemented by the teachers or by the researchers who conduct the evaluations. They can be applied to different types of domains. In the literature they have been generally applied in math or science domains such as statistics or physics (e.g., González‐Cabañes, 2020; Kapur, 2014; Newman, 2019; Schwartz, 2011), but also in other domains such as psychology, or pedagogy (e.g., Carnero‐Sierra, 2020; Glogger‐Frey, 2015; Schwartz, 1998). PS‐I methods have been applied successfully with students from age 12 through adulthood (e.g., González‐Cabañes, 2020; Kapur, 2014; Schwartz, 2011), and a few studies have used PS‐I methods with primary school children (e.g., Chase, 2017; Mazziotti, 2019). PS‐I interventions have also been conducted in collaborative contexts, in which groups of two or more students work on the initial problem‐solving activity (e.g., Glogger‐Frey, 2017; Kapur, 2012a), or in individual contexts, in which students work by themselves on the learning activities (e.g., Glogger‐Frey, 2015; González‐Cabañes, 2020). For the purpose of generalizing our findings broadly, in the upcoming review we will include studies conducted with students of any age, in any type of course, with collaborative or individual work, and with any kind of implementer.

There is also great potential variability regarding the intensity of PS‐I interventions. The duration of the initial problem‐solving phase generally spans between 12 min (e.g., González‐Cabañes, 2020) and 100 min (e.g., Kapur, 2012). The number of problems included in this initial activity can also vary, generally ranging between one (e.g., Glogger‐Frey, 2015; Weaver, 2018) and two (e.g., Chase, 2017; Glogger‐Frey, 2017). In regard to the times in which PS‐I is implemented within an intervention, generally PS‐I is only applied once for one specific lesson (e.g., Chase, 2017; Glogger‐Frey, 2017; González‐Cabañes, 2020; Kapur, 2012; Weaver, 2018), but there are other studies that applied it repeatedly over a longer time frame (e.g., Likourezos, 2017). Considering all these factors and the different potential durations of the explicit instruction phase, there are interventions with a great variety of total time invested.

It is just as important to consider the variability of interventions used as controls to compare the efficacy of PS‐I. It is often the case that PS‐I is compared with what are generally called ‘Instruction before Problem‐Solving’ interventions (I‐PS). Similarly than PS‐I, these interventions also include a problem‐solving phase related to the target contents, but only after students have received some explicit instruction about them. This initial instruction is often provided through lecture (e.g., Kapur, 2014; Weaver, 2018) or through worked examples to study (e.g., Schwartz, 2011). Worked examples refer to problems that show the resolution procedures. Interventions with no problem‐solving phase are also used, in which the initial problem‐solving activity of PS‐I is substituted with a worked example study (e.g., Glogger‐Frey, 2015; Glogger‐Frey, 2017). Lastly, other comparative interventions are more alike to the PS‐I interventions in the sense that they also start with the initial problem‐solving activity, but they provide some content guidance along with it. For example, it is common that some parts of the solution procedures are written (e.g., Likourezos, 2017; Newman, 2019). All of these types of comparisons will be considered within this review in as much as they have the same activities included in the instruction phase of the PS‐I intervention. Yet, separate meta‐analysis will be used for each type of comparison.

### 1.3 How the intervention might work

**Figure 1:**
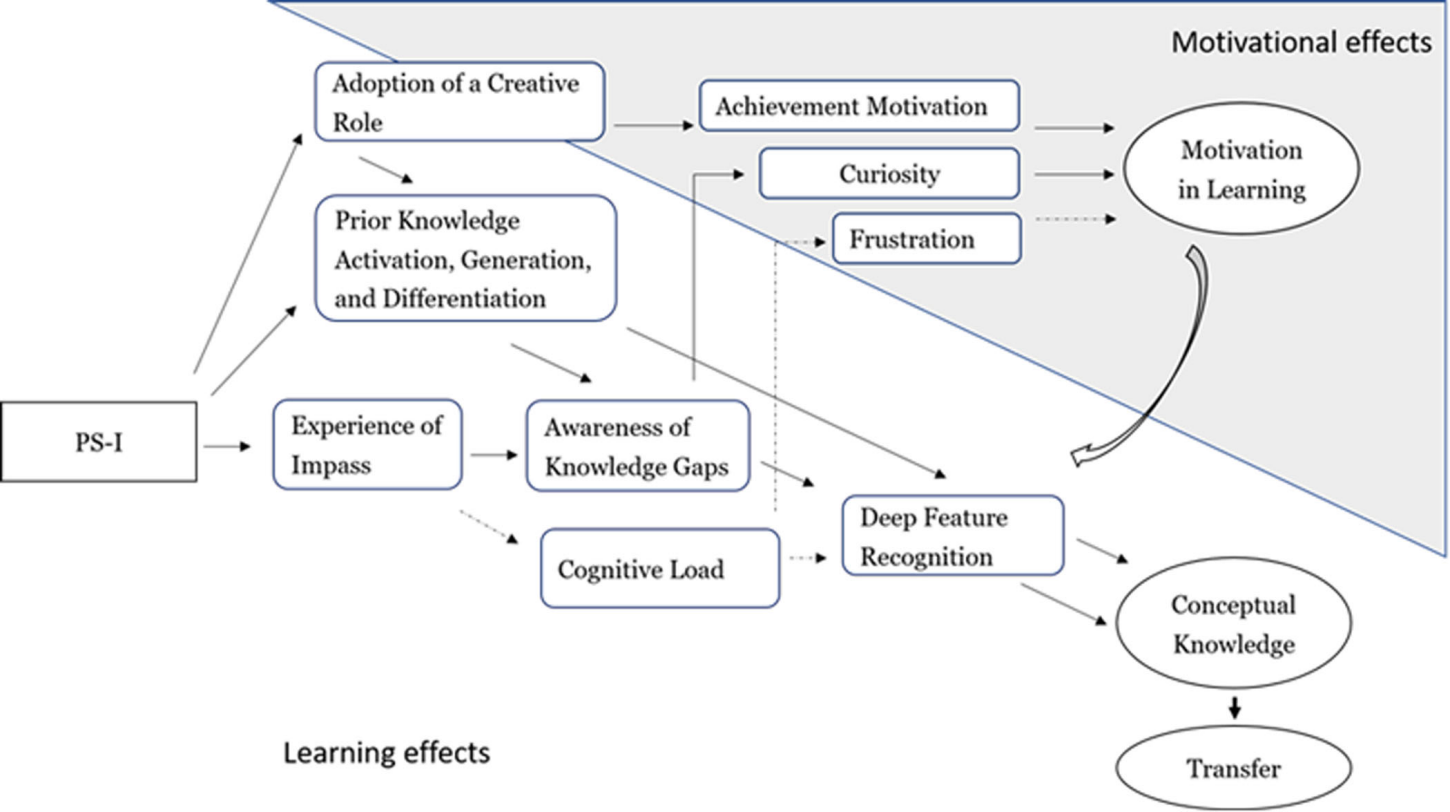
Theoretical model of different variables that might be associated with the efficacy of problem solving before instruction (PS‐I).

There are several mechanisms of PS‐I interventions that can influence learning and motivation, either positively or negatively. These mechanisms can interact, either compensating or reinforcing each other. Figure 1 depicts a proposal of these mechanisms, which is based on the theoretical proposal of Loibl (2017) regarding the cognitive PS‐I mechanisms that influence learning, but it aims to integrate motivational mechanisms within it.

#### 1.3.1 Potential PS‐I learning mechanisms

One potential mechanism through which PS‐I can favour learning is the opportunity in the initial problem‐solving activity to activate, differentiate, and generate prior knowledge in relation to the concepts that will be explained later (Kapur, 2012a). As students try to explore different solutions, they can become familiar with the problem situation and relevant features of the concepts to be explained. This familiarization can help students to more easily understand and integrate the explanations given later.

Furthermore, PS‐I also gives students a creative role during the exploration of the initial problem. Students can generate solutions using their own ideas, including ideas seen in previous classes, but also ideas from real life experiences. Ideas from real life experiences are accessible to all students and can constitute an additional and important support to integrated learning (Kapur, 2011a).

Several studies support the efficacy of activating prior knowledge with this creative component of generating personal ideas. Relative to other interventions where prior knowledge is activated through exploratory activities without this creative component, PS‐I led to greater conceptual understanding and transfer at the end of the lesson (Glogger‐Frey, 2017; Kapur, 2011; Kapur, 2014a; Schwartz, 2011). Also, it is interesting that the number of solutions generated by students during the initial problem‐solving phase of PS‐I have been associated with greater conceptual knowledge and transfer, regardless of whether the generated solutions were right or wrong (Kapur, 2011a; Kapur, 2012).

Another complementary mechanism of PS‐I that can favour learning is its potential to increase awareness of knowledge gaps. Humans often process information superficially, and unconsciously use this superficial knowledge to support a false illusion of understanding (Kahneman, 2011). In this regard, the experience of impasses within the initial problem can help students to become more aware of their knowledge gaps, which in turn can facilitate further exploration and recognition of deep features (Chi, 2000; VanLehn, 1999).

In support to these claims, several studies showed that students in PS‐I interventions reported higher awareness of knowledge gaps than students in alternative interventions in which the new concepts were directly explained from the beginning (Glogger‐Frey, 2015; Loibl, 2014). Also, students in PS‐I interventions had a better memory of the structural components of the problems presented than students in other interventions where the same problems were presented after some explanations (Schwartz, 2011) or together with explanations (Glogger‐Frey, 2017).

As discussed in Loibl (2017), it is important to consider the synergy that can occur between these mechanisms. It is likely that the greater the creative role assumed by the students, the greater the number of solutions they try, and the greater their activation of prior knowledge. In turn, it is also likely that the greater number of solutions attempted leads to greater opportunities to make mistakes, find impasses, and become aware of their learning gaps (see diagonal arrows in Figure 1 towards conceptual knowledge). The process can also be recursive. As learners become more aware of their learning gaps, prior knowledge activation is also more likely to be related to relevant aspects of concepts.

Lastly, another mechanism that can synergically reinforce these learning processes is the potential effect of PS‐I to increase motivation for learning, which is discussed in the following section. Motivation is defined as our desire to engage in the learning activity (Núñez, 2009), and can increase engagement in all learning processes previously mentioned (see wide white arrow coming out of Motivation for learning in Figure 1).

#### 1.3.2 Potential PS‐I motivation mechanisms

PS‐I can increase motivation for learning through several potential mechanisms (see horizontal arrows in Figure 1). First, it can be facilitated by the previously hypothesized PS‐I effect of promoting awareness of knowledge gaps. Some theories assume that we are intrinsically driven to acquire knowledge, and the mere perception of knowledge gaps often triggers curiosity, or the desire to fill that gap (Golman, 2018; Loewenstein, 1994). Several studies have shown that students who learned through PS‐I experienced greater curiosity and interest than students who started the learning process with explicit instruction (Glogger‐Frey, 2015; Lamnina, 2019; Weaver, 2018). Also, the study of Glogger‐Frey, 2015 found associations between the perception of knowledge gaps in PS‐I and curiosity.

The creative role that students can adopt in PS‐I can stimulate achievement motivation, which refers to the motivation to perform well in the learning activities (Núñez, 2009). Students in the initial problem‐solving activity of PS‐I are creating information, rather than just assimilating information given from outside, which can trigger a sense of responsibility and ownership in the task of constructing knowledge (Ryan, 2009; Urdan, 2006). Based on that, some students might try to perform the best they can to see their performance capabilities, and to maximize their learning. Literature in this sense is very scarce, but it was observed in one study that high school students in a PS‐I condition for learning geometry experienced higher achievement motivation than students in more guided interventions (Likourezos, 2017).

It is also important to consider the interrelation between learning and motivation. The hypothesized higher learning in PS‐I can lead to a higher sense of self‐efficacy for students in this condition, which in turn can increase motivation for learning (Núñez, 2009).

#### 1.3.3 Potential negative effects of PS‐I on learning

Although the experiencing of impasses can have important benefits when triggering awareness of knowledge gaps, it might have some negative implications. Given the inexperience of students in the topic, it is likely that they spend time attending to irrelevant information when they try to solve the impasses. In turn, this can generate extraneous cognitive load (Clark, 2012), which refers to a saturation in our attentional capacity due to processing irrelevant aspects, and can reduce the attentional resources available to focus on important information (Sweller, 2019). Therefore, this extraneous cognitive load can interfere in the recognition of deep features during the initial problem (Clark, 2012).

In this regard, several studies have shown that students in the initial problem‐solving activity of PS‐I reported higher extraneous cognitive load than students who faced similar problems that included explanations of the solution processes (Glogger‐Frey, 2015; Glogger‐Frey, 2017; Likourezos, 2017). In turn, this higher extraneous cognitive load was associated with lower learning (Glogger‐Frey, 2015; Glogger‐Frey, 2017), even when the final learning was higher within students in the PS‐I condition (Glogger‐Frey, 2017). Overall, these results suggest a complex interaction of negative and positive effects in PS‐I interventions, in which the positive mechanisms that we have described is balanced with the extraneous cognitive load that some students can experience (see the dashed arrow coming from extraneous cognitive load towards recognition of deep features in Figure 1).

#### 1.3.4 Potential negative effects of PS‐I on motivation

A potential factor that can demotivate students in PS‐I interventions is the frustration they can feel in the initial problem‐solving phase (Clark, 2012). Frustration can arouse in the initial problem‐solving activity because of the experiencing of extraneous cognitive load or because of the sensation of failing to achieve the correct solution within the impasse experiences. In turn, frustration can have demotivating effects, such as reducing intrinsic motivation (Loderer, 2018), or contributing to fatigue (Pekrun, 2011; Pekrun, 2012). Recent studies have shown higher frustration (González‐Cabañes, 2020) or negative affect (Lamnina, 2019) in PS‐I students versus students in a typical instruction condition where they started the learning process with explanations.

#### 1.3.5 Hypotheses about general effects

Considering this variety of potential positive and negative mechanisms, it is of great importance to study the final effects in learning and motivation. In line with the previous reviews of Loibl (2017) and Sinha (2021), we expect that PS‐I, in comparison with alternative interventions, will be associated with the higher performance of students in post‐tests of conceptual knowledge taken after the lesson, but not with performance in concurrent post‐tests of procedural knowledge. Procedural knowledge can be acquired through memorization, and therefore the potential described PS‐I mechanisms of promoting activation of prior knowledge and awareness of knowledge gaps might have little influence on it. Yet, we expect that these potential mental processes can greatly impact conceptual knowledge, which refers to the understanding of principles and relationships that underlie concepts and procedures. Conceptual knowledge not only relies on memorization, but also on the identification of structural features of the concepts. These mental processes can also have a great impact in transfer. Transfer can be facilitated by the activation of prior knowledge, as it can rely on the integration between prior knowledge and new knowledge (Loibl, 2017), and generally by the acquisition of conceptual knowledge (Mayer, 2003). Only with a clear mental representation of how the procedures work can we perceive whether the procedures generalize to other contexts.

Although there is no previous review regarding motivation for learning, we expect that PS‐I interventions will be associated with higher scores in self‐reports of interest taken after the lesson, because of the effects PS‐I can have on achievement motivation and curiosity.

#### 1.3.6 Factors that can moderate the efficacy of PS‐I

In spite of these hypothesized general effects of PS‐I on learning and motivation, a great variety of factors can moderate these effects. Among them, are design features of the interventions, intensity of the interventions, age of students, learning domain, and activities used as control. It is important to note that, as previoulsy described, learning and motivation can benefit each other, and therefore we will consider all moderators as potentially influencing both.

##### Design features

The different design features used in PS‐I interventions can have different effects on the previously described mechanisms, and therefore in the general efficacy of PS‐I on learning and motivation. Below we describe some of the design features frequently discussed and used in the literature, which we will consider as potential moderators in the present review.

- *Contrasting cases*. Contrasting cases is a form of guidance that is often used to present the data of the initial problem (e.g., Loibl, 2020; Schwartz, 2011). It consists of examples that differ on a few features that are relevant to the target knowledge (Schwartz, 2004). For example, in the contrasting cases shown in Section 1 of Table 1, which was designed for learning about statistical variability of data distributions, we can see how the distributions of scores for player A and player B differ in the range, but not in other features of the distribution such as the mean, the number of scores, and the spread of scores. In contrast, the distribution of player B and player C's scores differ in the spread of the scores, but not in the range or other characteristics. It has been argued that the comparison of cases can help students focus on the relevant features of the problem (Loibl, 2017; Salmerón, 2013; Schwartz, 2004). Also, contrasting cases can help students become aware of their knowledge gaps, because students can rank the cases and self‐evaluate their solutions in regard to it (Loibl, 2017; Schwartz, 2004). Finally, contrasting cases may reduce extraneous cognitive load during problem‐solving and its associated frustration.
- *Metacognitive guidance*. The initial problem is often presented with metacognitive guidance during problem solving (e.g., Holmes, 2014; Roll, 2012). Metacognitive guidance refers to prompts that do not address content, but rather are meant to stimulate conscious mental strategies such as monitoring and reflection processes. This type of guidance can stimulate mental processes that can lead students to become more aware of knowledge gaps and to recognize deep features (Holmes, 2014; Roll, 2012). For example, the metacognitive guidance in Section 2 of Table 1 can trigger students to reflect on critical features they perceive in the data distributions and the limitations of the solution ideas they generate.
- *Collaborative work*. Allowing students to work on the initial problem‐solving activity in small groups, rather than asking them to work individually, might influence the relative efficacy of PS‐I (Mazziotti, 2019). Collaborative problem‐solving is a context that brings opportunities for elaborating and critiquing ideas (Kapur, 2012a; Webb, 2014). Several studies have found that problem‐solving in pairs was associated with higher performance than working individually (Teasley, 1995; Webb, 1993). Also, the extent to which students engage in dialectical argumentation with each other's ideas and explain their problem‐solving strategies has been associated with higher problem‐solving achievement and higher acquisition of conceptual knowledge (Asterhan, 2009; Webb, 2014). Based on this, we expect that working on the initial problem in groups will be associated with higher efficacy for PS‐I than working individually.
- *Building explanations of explicit instruction phase on students' solutions*. PS‐I interventions often include explanations in the explicit instruction phase that draws students' attention to the affordances and limitations of students' typical solutions given in the previous problem‐solving phase (e.g., Kapur, 2012a; Kapur, 2014; Loibl, 2014a). An example can be seen in Section 3 of Table 1. It can be considered a form of guidance for the problem‐solving activity that, rather than given during the problem‐solving phase, it is provided afterwards to help students reorganize the ideas they activated during the problem‐resolution. It has been argued that this feedback can help students to become more aware of their knowledge gaps, and to focus on the relevant features within the complexity of the target concepts (Kapur, 2012a; Loibl, 2017).

##### Duration of the PS‐I intervention

The duration of the PS‐I intervention can have an important effect on its efficacy to promote learning and motivation, because of a higher dosage of the hypothesized PS‐I effects. We expect a higher efficacy of PS‐I in longer interventions.

##### Age of students

As considered in the prior review of Sinha (2021), age might be associated with the relative efficacy of PS‐I because of the relation between age and metacognitive development. Metacognition refers to the awareness of our own mental processes and the control we have over them (Schraw, 1994), and has been argued to have an important influence on several PS‐I mechanisms (Glogger‐Frey, 2017). First, it can help students to become aware of their knowledge gaps. Students with low metacognitive skills might not relate the limitations of the solutions they generated with the solutions explained later (Roll, 2012). Second, metacognition can also help students to discern what information is relevant from the information that is not, which can reduce the extraneous cognitive load experienced during the initial problem‐solving phase and its associated frustration. However, these metacognitive capacities might develop slowly with age (Veenman, 2005). Based on these assumptions, we expect that the higher the age of the students, the higher the efficacy of PS‐I to promote conceptual knowledge, transfer, and motivation for learning.

##### Learning domain

The learning domain in which PS‐I is applied might have an important influence on the efficacy of PS‐I. In math and science domains (e.g., statistics, physics) conceptual structures are often abstract and complex, and the deep learning processes expected to be promoted in PS‐I interventions might be more significant in these domains. Specifically, in this review we expect that higher efficacy of PS‐I will be found in math and science domains than in other domains.

##### Control conditions used for comparison

The types of control conditions used to compare PS‐I can have a great influence in the relative efficacy of PS‐I. In the literature we have identified several types of comparative interventions:

1. *Instruction with lecture before problem‐solving*. Share with the PS‐I intervention both the problem‐solving phase and the other learning activities in the instruction phase, but instead of introducing the contents with the problem‐solving activity, the contents are introduced with a lecture about the target concepts, in which students have to listen to the explanations of the concepts at a given pace.
2. *Instruction with worked‐examples exploration before problem‐solving*. Share with the PS‐I intervention both the problem‐solving phase and the other learning activities in the instruction phase, but instead of introducing the contents with the problem‐solving activity, the contents are introduced with a worked example that they study at their own pace.
3. *Instruction with worked example exploration before further instruction*. These interventions do not include problem‐solving activities. They share with the PS‐I intervention all the learning activities in the instruction phase, which are activities that do not include problem‐solving. Also, instead of the initial problem‐solving activity of PS‐I, they start with a worked example, where students study the resolution procedures to problems at their own pace.
4. *Problem‐solving with content guidance before instruction*. Share with the PS‐I intervention all the learning activities in the instruction phase, and also start with the initial problem‐solving activity used in the PS‐I intervention, but provide students with some content guidance during it.

We expect that PS‐I would lead to higher benefits in terms of learning and motivation than these control conditions. Although extraneous cognitive load and frustration might be lower in the initial phases of these control conditions than in PS‐I, the initial problem‐solving phase of PS‐I gives students an opportunity to acquire a creative role and to experience impasses that are not given in other conditions.

Nevertheless, we expect that the relative efficacy of PS‐I would be higher when compared with conditions that introduce the concepts with a lecture (1), rather than when they are introduced with worked‐examples or problems with content guidance (2‐4). In these latter control conditions, students start by exploring information at their own pace, which, similarly to PS‐I, gives them the opportunity to activate prior knowledge before receiving explanations from the professor. As we have previously described, activation of prior knowledge can potentially favour the assimilation of explanations (Carriedo, 1995; Smith, 1992; Sweller, 2019). This pattern of results would be in line with the results of Newman, 2019, in which the advantage of PS‐I in terms of learning was higher when compared against the introduction of concepts with a lecture, rather than when PS‐I was compared against interventions that introduced concepts with worked‐examples or problems with content guidance.

Additionally, among these last comparative interventions (2‐4) we also hypothesize that the relative efficacy of PS‐I will be higher when compared with interventions that do not include a problem‐solving phase (3) than when this problem‐solving phase is included (2, 4). Problem‐solving activities can help students to reflect and reason about the target concept, and missing these types of activities can have implications in conceptual knowledge acquired.

### 1.4 Why it is important to do this review

There are four reviews in the literature that have provided interesting insights into the efficacy of PS‐I (Darabi, 2018; Jackson, 2021; Loibl, 2017; Sinha, 2021).

First, the review by Jackson (2021) is a qualitative review that included studies from educational databases and conferences that addressed factors that can influence learning from failures in STEM courses. It included studies about PS‐I interventions, where failure is expected in the initial problem, but also other type of studies that addressed learning from failures. Considering the results of the 35 papers included, they discussed that the efficacy of learning from failure could depend on factors such as whether students conceptualize the failures as learning opportunities, the promotion of positive affective reactions such as persistence, the promotion of a classroom climate to speak about failures and embrace them, and the use of failures as stimuli to identify misconceptions and induce thoughtfulness about key features. Although the presence of these factors can be important in the efficacy of PS‐I to promote learning and motivation, this review was limited in that these factors were not addressed quantitatively.

The review of Loibl (2017), specifically focused on the efficacy of PS‐I to promote learning, and provided a vote counting procedure to synthetize the literature results. Their results suggested that the efficacy of PS‐I depended on the type of learning outcome considered. Across the 34 studies they identified, they found that most studies reported no significant differences between PS‐I and other alternative approaches in terms of procedural knowledge, which just refers to the capacity to reproduce memorized procedures covered in class. However, when the evaluation was made in terms of conceptual knowledge or transfer, PS‐I generally led to more positive results. For example, out of 17 studies, they identified 10 studies where transfer was significantly higher in PS‐I approaches, 1 study in which it was higher in alternative approaches, and 6 studies showing no significant difference. They also explored the effect of different PS‐I design features, for which they proposed an interesting moderator: whether PS‐I was presented in combination with techniques oriented to foster awareness of knowledge gaps and recognition of deep features, such as contrasting cases or building instruction on students' solutions. They found that when any of these forms of guidance were present, it was more likely to find a positive significant difference for PS‐I.

However, there are important aspects in relation to the scope and methods of this review that are important to address. First, the results were analysed using a vote counting procedure instead of meta‐analysis techniques. Second, their results could be easily contaminated by publication bias and availability bias, because, beyond looking into the list of references of some of the localized studies, they did not try to find studies within the grey literature. Finally, they did not consider the outcome of motivation for learning, nor some of the other potential moderating factors commented here, such as the type of control activities used for comparing PS‐I, or the intensity of PS‐I interventions.

The review by Darabi (2018) provided some meta‐analytic evidence about the general efficacy of PS‐I on learning. However, the scope of this review was larger and less specific. Their goal was to evaluate educational approaches based on learning from failures, which included PS‐I approaches, but also other failure‐driven approaches. In spite of that, out of the 23 studies that they ended up identifying, 22 were about the effectiveness of PS‐I. The results suggested that students who learned through failure‐driven approaches acquired more knowledge than students who learned through alternative approaches, with a moderate effect size (*g* = 0.43). They also explored the influence of interesting moderators such as age or intensity of the intervention, for which they found no significant results.

Nevertheless, this review has important aspects to address. Beyond mixing results of PS‐I interventions with other failure‐driven interventions, results can be biased because of mixing different types of learning outcomes. They aggregated together outcomes that referred to procedural knowledge, conceptual knowledge, and transfer, which according to the review of Loibl (2017) can lead to very different results. Also, these results might be biased by availability and publication bias, as suggested by their post‐hoc analyses. They only searched in few databases and using a very short variety of keywords, which can explain why, in spite of being more recent than the Loibl (2017) review, they identified considerably fewer studies.

Lastly, the review of Sinha (2021), specifically focused on the efficacy of PS‐I interventions to promote learning in comparison to other interventions in which explicit instruction were provided from the beginning. Up to date, their review is the one that includes more studies about this topic. Sinha and Kapur (2021) included 53 studies that were selected from studies that had cited in Google Scholar some of the reference articles of productive failure, a specific approach of PS‐I. Their review also had the advantage of analysing the results with meta‐analysis techniques. The results showed a significant moderate effect in favour of PS‐I (*g* = 0.36) versus alternative interventions, using an aggregation of measures that included tests of conceptual knowledge and transfer. Their moderation analyses showed that this effect was higher when using PS‐I design features such as using group work in the problem‐solving phase (*g* = 0.49), or building the explanations of the explicit instruction phase on students' solutions (*g* = 0.56). The use of other design features, duration of the interventions, age, and learning discipline did not show significant differences.

However, it is important to consider some aspects to complement in this review. First, the search of studies was limited to studies citing pioneer papers of productive failure in Google Scholar, which can leave behind studies about PS‐I not available in this source, or that are disconnected from the productive failure literature. Second, it did not consider the different types of control interventions to compare the efficacy of PS‐I. Also, it did not include motivational outcomes. Lastly, most of the effect sizes reported were based on a substantial body of studies in which no equivalence between conditions was kept in terms of learning materials. Only the effect size they reported for the subgroup of experimental studies is expected to be free from these studies (*g* = 0.25).

While trying to overcome their mentioned methodological limitations, the upcoming review aims to update the evidence of these four reviews. It also aims to consider a greater variety of outcomes and moderators. Regarding the outcomes, rather than just considering different types of learning, the upcoming review will also consider motivation for learning. Regarding factors that can moderate the efficacy of PS‐I, rather than just considering different PS‐I design features, and contextual factors such as duration or learning domain, it will also consider the different types of control activities used to compare the efficacy of PS‐I. Lastly, it will also provide separate results for the main analyses, in which equivalence of materials between PS‐I and other interventions is maintained, and additional exploratory analyses, in which such equivalence is not necessarily maintained.

Results of this review can have important implications when considering whether or not to introduce PS‐I into the educational practice. The use of PS‐I is very scarce (Pan, 2020), and it is important to offer updated evidence of whether it can contribute to the promotion of motivation, conceptual knowledge, and capacity to transfer learning. This evidence can help instructors to reduce the uncertainty of trying it. Also, it can help them to get guidance about which design features or contextual factors can contribute to its efficacy.

## 2 OBJECTIVES

The purpose of this review is to synthesize the evidence about the efficacy of PS‐I to promote learning and motivation in students. Specifically, this review is designed to answer the following questions:

- To what degree does PS‐I affect learning and motivation, relative to alternative learning approaches?
- To what extent is the efficacy of PS‐I associated with the use of different design features within it, including the use of group work, contrasting cases, and metacognitive guidance in the initial problem‐solving activity, and the use of explanations that build upon students' solutions in the explicit instruction phase?
- To what extent is the relative efficacy of PS‐I associated with the contextual factors of activities used as control, age of students, duration of the interventions, and learning domain?
- What is the quality of the existent evidence to evaluate these questions in terms of number of studies included and potential biases derived from publication and methodological restrictions?

## 3 METHODS

### 3.1 Criteria for considering studies for this review

#### 3.1.1 Types of studies

All studies included in the review will fulfil the following requirements:

- They must involve a comparison of at least one group that goes through PS‐I with at least one comparative group that goes through an alternative intervention in which the teaching of the target concepts starts by providing students with some content.
- They will either be randomized controlled trials or quasi‐experimental designs in which different students are assigned to the PS‐I conditions and the control conditions. For both types of designs, we will include studies in which the unit of assignment is either the students or students' groups (e.g., class groups, work groups). Also, for both types of designs, we will include studies in which the assignment method is random, quasi‐random, or even not random.

Nevertheless, we will exclude studies if the assignment leads to any difference between the PS‐I group and the comparative group that can affect learning (e.g., if one group belongs to class groups or schools with recognized better performance than the other group), or if pre‐existing differences between these two groups in terms of age, gender, or previous knowledge are statistically significant, as indicated by inferential statistical tests for group comparisons, using a level of statistical significance of *p* ˂ .05. This exclusion criterion would apply for both quasi‐experimental designs and randomized controlled trials. Studies where teaching time is not the same for both groups will also be excluded.

In regard to the equivalence of teaching contents, we will have different inclusion criteria for the main analyses and complementary exploratory analyses. For the main analyses, we will only include studies in which the PS‐I group and the control group receive the same contents about the target concepts, and in which the learning activities are also the same but with the exception that the PS‐I group would perform a problem‐solving activity at the beginning of the intervention, and, during the same amount of time but not necessarily at the same time, the comparative group would perform alternative activities covering the same contents.

For the additional exploratory analyses, we will include studies in which such an equivalence of contents and activities is not maintained, which often occurs in studies that use a business‐as‐usual comparative condition. For example, in some studies the explicit instruction phase of the PS‐I condition includes explanations that build on the students’ generated solutions, while such explanations are not given in the comparative condition (e.g., Kapur, 2012a).

#### 3.1.2 Types of participants

The studies must have child or adult students as participants. We will not have an exclusion criterion based on age. Students from any developmental stage can potentially benefit from PS‐I. Eligible samples would also include populations at risk because of socio‐economic disadvantage, such as students from specific ethnicities or minorities, inner‐city schools, prison populations, or students who have poor school performance. These populations are important for inclusive education policies, and all of them have the potential to benefit from PS‐I. Samples consisting exclusively of people with a specific psychological diagnosis will be excluded because of the complexity of interpreting the variability generated by these populations.

#### 3.1.3 Types of interventions

Studies eligible for this review will have to examine the effectiveness of PS‐I, and therefore will be required to have at least one group of students go through this approach, which will be defined by the following components:

- Students start the learning process with a problem‐solving activity that targets concepts they have not yet learned,
- For which they are given time to develop solutions on their own,
- And that will be followed by a separate phase of explicit instruction about the new concepts, in which students can listen to or read these concepts.

Within PS‐I interventions there are possible additional characteristics that we might consider in the moderation analyses, but not within our inclusion criteria. Specifically, for the initial problem‐solving phase we will consider the presence of (a) contrasting cases; (b) metacognitive guidance; and (c) collaborative work. For the posterior instruction phase we will consider the presence of explanations that build upon students' solutions. Table 1 shows examples for these variables. We will also consider interventions with different durations, which can range from one session to several sessions.

It is important to note that we will exclude studies where students are faced with novel problems but they are not given the opportunity to solve them on their own. Examples of this situation include studies where students have access to external sources of information from the beginning of the problem‐solving activity (e.g., Tandogan, 2007), or where the problem only acts as a scenario to stimulate students' expression of their first intuitions.

Regarding the comparison condition, eligible studies have to at least include one control group that is given the same learning materials as the PS‐I group, but instead of going through the initial problem‐solving activity, they work through an alternative activity. Examples of these alternative activities include: (a) the same problem‐solving activity but used as a practice activity, which is provided to students once they have received all or part of the instruction about the target concepts; (b) the same problem‐solving activity but used in the form of a worked example; (c) other alternative activities that maintains a balance between the two interventions in terms of time and content covered.

Examples of eligible studies are summarized below:

- Study 1 in Kapur (2014) assigned several statistics classes, composed of 75 9th grade students, into two learning conditions. In the PS‐I condition, students first had one hour to solve a novel problem about designing variability measures (phase 1), then they received another hour of explicit instruction about the standard deviation concept accompanied with practice problems (phase 2). In the control condition, students went through the same two phases but in reverse order. In the post‐test, students in the PS‐I condition outperformed those in the control condition in conceptual knowledge and transfer of learning, but not in procedural knowledge. During the learning process, students' engagement did not differ between conditions, but mental effort was higher for students in the PS‐I condition.
- A study by Likourezos (2017) assigned, within their classes, 72 8th grade students into three learning conditions that spanned six 1‐h sessions of geometry. In the PS‐I condition the sessions were composed of two phases, a 30 min phase in which students solved novel problems, then an explicit instruction phase of 30 min. In the control condition the initial problems were substituted by worked out examples, which were the same as the problems used in the PS‐I condition but totally solved and included explanations that students could study. In a second control condition, which authors called partially‐guided, these worked‐examples only included the final solution, and students had to figure out the process. Post‐test results showed no significant differences between conditions in learning transfer nor procedural knowledge. Yet, some differences were found during the learning process in terms of motivation and the cognitive load students experienced.

#### 3.1.4 Types of outcome measures

##### Primary outcomes

Eligible studies must report either outcomes for learning or motivation for learning after the intervention.

In terms of learning we will consider two of the primary outcomes already analysed in the review of Loibl, 2017, conceptual knowledge and transfer:

- Conceptual knowledge is defined as the understanding of the structure and relationships that underlie a taught concept or procedure. It is generally measured by testing students in principle‐based reasoning, where they have to explain the why of different situations or procedures, or in the ability to connect different representations (refer to the conceptual knowledge post‐test in Kapur, 2012 for an example).
- Transfer is defined as the ability to adapt the learned concepts to new situations. It is generally measured by asking students to solve problems that have no explicit relation with the concepts learned, and that are novel in the sense that have a new structure or occur in a new context compared to the problems students have previously seen (refer to the transfer post‐test in Kapur, 2012 for an example).

Measures of conceptual knowledge and transfer reported in the literature are generally not previously validated. They are generally created for the specific contents seen in each study. To be as comprehensive as possible we will include studies with un‐validated measures as long as the items correspond to our definitions of conceptual learning or transfer. We will only consider measures of students' performance, based on knowledge tests completed by students after the end of the interventions.

Concerning motivation, the planned primary outcome will be motivation for learning, which we define as a desire to engage in learning about the topic that has been taught. For its measurement we will primarily consider self‐report measures of interest, which refers to the perception of caring about learning the topic, and which is generally measured with questionnaires that ask students about the intensity with which they have different motives for learning. As a second priority to measure motivation for learning we will also consider self‐report measures of curiosity. Curiosity is an important component of interest, but it is more specific in the sense that refers to the desire of knowing. It is generally measured through questionnaires that ask students about the intensity with which they feel this sensation. The PS‐I literature has often used measures of interest or curiosity that have not been previously validated. To be as inclusive as possible, studies with non‐validated measures will be considered, but only as long as the items correspond with our definition of motivation for learning. Measures of engagement in the learning task will not be considered as indicators of motivation for learning, because engagement can be influenced by different factors not related to motivation, such as the task requirements.

Finally, it is important to consider that in the literature these measures are often measured at different time points during and after PS‐I interventions. For the main analysis we will consider the first measurement taken at the end of the learning process. Yet, other measurement times might be considered if available for several studies.

##### Secondary outcomes

We will code, and potentially consider as secondary outcomes:

- Procedural knowledge, defined as the ability to correctly apply the learned procedures (Loibl, 2017). It is generally measured by testing students in the ability to carry out a set of steps, such as solving plug‐and‐chug problems, or questions to develop a set of learned procedures.
- General measures of learning that mix items of procedural knowledge, conceptual knowledge, and/or transfer. These types of measures can be common in applied studies that use a typical exam to evaluate performance.
- Factors that can influence the learning process, such as engagement, cognitive load, or number of solutions generated during the problem‐solving activity.
- Factors that can influence motivation, such as self‐efficacy, anxiety and frustration.
- Outcomes related to implementing the activities, such as work load experienced by the professors who implement the activity.

### 3.2 Search methods for identification of studies

Different sources will be searched to include published and unpublished international studies written in English or Spanish, with no date restriction. Although we might have problems scanning studies written in other languages than English or Spanish, no language limits will be applied in the searches.

#### 3.2.1 Electronic searches

Based on the guidelines and lists of databases of Kugley (2017) for selecting electronic searches, we will search within the following sources that include journal articles, conference proceedings, government documents, and dissertations:

- Databases for scientific literature, either with a general scope or with a scope focused on education. Across them, we will search in all the six indexed of Web of Science, PsycINFO, ERIC, MEDLINE, Google Scholar, Academic Search Complete (EBSCO), Education Abstracts (EBSCO), Education Full Text (EBSCO), SciELO, and Dialnet.
- Databases that are broadly open to grey literature. Across them, we will search in EBSCO Open Dissertations, ProQuest Dissertations & Theses Global, EThOS, TESEO, and the Networked Digital Library for Theses and Dissertations.

Within these databases, we will use a combination of keywords that refer to PS‐I interventions (e.g., ‘Problem‐solving’ AND ‘Explicit instruction’ OR ‘Problem‐solving before Instruction’ OR ‘Productive Failure’ OR ‘Inventing to Prepare for Future Learning’). To make the output more specific, this combination may be restricted with a combination of keywords that refer to our primary outcomes (e.g., ‘learning’ OR ‘motivation’) and/or a combination of keywords referring to our eligible population (e.g., ‘students’ OR ‘pupils’). Appendix 1 shows an example of a strategy search in PsycINFO.

#### 3.2.2 Searching other resources

Beyond electronic searches, we will use other sources, including:

- Citations associated with systematic reviews and relevant studies. Specifically, we will search in the list of references of previous systematic reviews about PS‐I (Darabi, 2018; Jackson, 2021; Loibl, 2020; Sinha, 2021). Additionally, we will use Google Scholar to search across the documents that have cited either these reviews or the reports that are considered pioneers in PS‐I approaches (Kapur, 2008; Kapur, 2012a; Schwartz, 1998; Schwartz, 2004). Lastly, the review team will check reference lists of included studies, and the citations in Google Scholar to these included studies.
- Conference proceedings of educational conferences. We will search in proceedings of conferences celebrated in the last 15 years of the European Educational Research Association (EERA) and the International Society of the Learning Sciences (ISLS).
- Documents from international and national organizations. We will search for publications in the OECD (https://www.oecd-ilibrary.org/), the UNESCO (https://www.unesco.org/es/ideas-data/publications), the World Bank (https://www.worldbank.org/en/research), the Eurydice Network (https://eacea.ec.europa.eu/national-policies/eurydice/), the US Department of Education (https://www.ed.gov/), and the Spanish Department of Education and Professional Training (https://www.educacionyfp.gob.es/portada.html).
- Hand searches of journals. Four journals that frequently publish about PS‐I interventions will be hand searched for documents published in the last 5 years: *Instructional Science*, *Learning and Instruction*, *Cognition and Instruction*, and *Journal of Educational Psychology*.
- Communications with international experts. After finishing the search in other sources, we will email all the contacting authors of the identified studies to ask them about additional studies they may know of, including unpublished studies. This email will contain a comprehensive list of the included articles along with the inclusion criteria.

### 3.3 Data collection and analysis

#### 3.3.1 Selection of studies

Study selection will be done through the software Covidence. After eliminating duplicated manuscripts, we will screen the titles and abstracts of the remaining manuscripts to evaluate their potential inclusion. Among these pre‐selected manuscripts, we will screen the full texts to consider if they meet our inclusion criteria. For these two screening processes, 20% of the manuscripts will be screened individually by two members of the team. If for a given subset of manuscripts, the level of agreement is below 80%, the subset will be screened again until reaching this standard. The level of agreement will be reported. Disagreements will be resolved by discussion until reaching consensus. If disagreements persist, a third reviewer in the team will be consulted.

#### 3.3.2 Data extraction and management

Data of the primary studies will be directly introduced in two forms in a Microsoft Access document that can be found in the following link, together with the coding manual: https://www.dropbox.com/sh/u3nr12ayilaezps/AADQngLciNF_gGLrRSpqKtofa?dl=0.

The first form is the Reports Form, and will be used to code information about each report that, after screening, contains any study suspected to be included in the review. It includes variables related to the following:

- Title of the report.
- Year of publication and type of publication.
- Authors and affiliations.
- Studies contained in the report.

The second form is the Studies Form, and will be used to code information of each study in the reports that has been preliminarily accepted to inclusion. It includes variables related to the following:

- Setting (e.g., public vs. private institutions, special education units, topic taught).
- Sample characteristics (e.g., sex ratio, age mean).
- Design features of the PS‐I and comparative interventions (e.g., use of contrasting cases, group work, metacognitive guidance).
- Information related to risk of bias (e.g., assignment procedures, control of extraneous variables, attrition)
- Implementation characteristics (e.g., person who delivers the intervention, duration of intervention).
- Types of control groups being compared.
- Characteristics of measures used (e.g., internal reliability).
- Characteristics of the effect sizes (e.g., time passed from the end of the intervention to the measurement).
- Effect sizes for different subgroups (e.g., effect sizes of subsamples with different levels of prior knowledge).

This form automatically calculates effect sizes and their related statistics after introducing the means, standard deviations, and sample sizes reported in the primary studies. In cases where this information is not reported, the coders will use the Campbell Collaboration online calculator to calculate effect sizes from other reported values.

To evaluate the coding reliability, the Studies Form will be completed by two coders for a random selection of 10% of the studies. Discrepancies will be resolved by further review of the reports and by discussion until an agreement is reached. If we identify relevant variables during the coding process, they will be added to the questionnaire.

#### 3.3.3 Assessment of risk of bias in included studies

Risk of biases will be assessed using several items in the Studies Form (refer to Data extraction and management) that address the five domains of the Cochrane Risk of Bias Tool for Randomized Trials (Sterne, 2019). However, in comparison to this tool, we changed some items in each domain to specifically adapt to the context of this review:

- Randomization process: we will code for whether the units of assignment are students or students' groups, and whether assignment is random. We will also code the identification of baseline differences in terms of gender, age, previous knowledge, or other relevant variable identified, and whether this data is reported.
- Deviations from the intended intervention: we will code for whether the PS‐I interventions and the control interventions were implemented in the same place, at the same time, with the same implementers, with the same durations, with similar levels of attrition, and covering the same contents. It will also be coded whether implementers were blind, and whether it was used a pre‐test including problem‐solving activities related to the contents to cover, which can create a PS‐I effect in the control interventions and therefore contaminate the results (Newman, 2019).
- Missing outcome data: missing data higher than 5% for any relevant comparison will be identified.
- Measurement of the outcome: appropriateness of the measure will be coded regarding whether the items correspond to the definition of the construct, using a Likert type scale (yes, probably yes, probably no, no, cannot tell). Other factors that will be coded include whether the measure was previously validated, and reliability indicators in terms of internal reliability and inter‐rater reliability.
- Selection in the reported result: the coder will assess the probability that the reported assessments or analyses were selected on the basis of the findings, using a Likert type scale (yes, probably yes, probably no, no, cannot tell).

For each of these five dimensions, coders will assess the degree of risk of bias (low, high, or some concerns). In case of assessing them as ‘high’ or ‘some concerns’, they will describe the specific effect sizes affected by this judgement, the direction in which the potential bias is suspected to affect (favours experimental, favours comparator, towards null, away from null, unpredictable), and the reasons behind it.

After evaluating these questions, coders will re‐evaluate whether some or all effect sizes taken from the study should be analysed according to the inclusion criteria. They will also classify these effect sizes into three categories referring to the general risk of biases: low, some concerns, or high.

- Low risk of bias will be assigned for studies in which are fulfilled two requirements: a) participants are randomly assigned to conditions (unit of assignment is the participant and the method of assignment was totally random), and b) there is enough information to assume equivalence between groups and interventions.
- Some concerns for risk of bias will be assigned for studies in which only one of these two requirements are fulfilled.
- High risk of bias will be assigned for studies in which none of these two requirements are fulfilled.

In case of selecting options ‘high’ or ‘some concerns’, descriptions about the specific effect sizes affected by this assessment, the direction of the potential bias, and the reasons behind it will be added.

#### 3.3.4 Measures of treatment effect

For the three primary outcomes of conceptual knowledge, transfer, and motivation for learning, we will use standardized mean difference effect sizes, or Cohen's *d* (Cohen, 1988), to estimate the effect of PS‐I interventions in comparison with other interventions used as a control, as indicated in the following formula:

$$d=\left(Mean_{\mathrm{PS}-I}-Mean_{\mathrm{Control}}\right)/s_{p},$$

where the numerator is the difference of the PS‐I group mean minus the control group mean, and the denominator is the pooled standard deviation for the two comparison groups. Larger effect sizes will represent a higher quantity of the outcome in the PS‐I group in comparison to the control group. Once these effect sizes are obtained, they will be adjusted with the small‐sample correction factor to provide unbiased estimates (Hedges, 1981), and 95% confidence intervals will be calculated from them.

#### 3.3.5 Unit of analysis issues

To prevent the inclusion of the same effect size twice in one analysis, effect sizes for different constructs and different evaluation moments would be analysed separately. In cases where one study provides more than one measure for one of the constructs we have defined, we will select only one measure. First, for that selection we will follow the priorities already specified for the primary outcomes (refer to Primary outcomes). Second, if the possibility to select two outcomes remains, we will select a previously validated measure over a non‐validated measure. Last, if the possibility to select several outcomes remains, we will select the measure that is most similar to those used by the other studies.

To prevent that a study that has been published in several reports is included several times in the analyses, at the end of the coding process we will explore nonobvious duplicates by looking for repetitions within the categories of key variables such as authors, date of publication, or effect sizes.

givenNamesIn multi‐aim primary studies that compare two PS‐I groups with one control group, we will carry two options following recommendations in Higgins, 2019 to avoid weighting as twice the values of the control group in the aggregated analyses: (a) when the two PS‐I groups are similar, we will treat them as a single group; (b) when they are not similar, the sample size of the control group will be divided in half before being compared with the PS‐I groups. A similar strategy but in reverse order will be conducted when a study compares one PS‐I group with two control groups.

##### Clustering issues

In the PS‐I literature it is common that the units of assignment to conditions are not the students, but clusters of students, either class groups or working groups (pairs or small groups of students that work together in the interventions). To correct for the artificial reduction in the standard error of the effect size estimates due to these clusterings, we will follow the recommendations in Higgins (2019) of multiplying the standard error by the square root of the ‘design effect’, whose formula is

1+(averageoftheclustersize–1)×intraclustercorrelationcoefficient.

For studies in which the intracluster correlation coefficient is not reported, we will use the coefficient of similar studies included in the review.

#### 3.3.6 Dealing with missing data

To deal with missing data, authors from primary studies will be contacted via email. In case the requested information is not received, the study will be reported, but the effects for which there is missing data will not be included in the analyses.

#### 3.3.7 Assessment of heterogeneity

We will evaluate the variability across studies using the *Q* statistic and its associated chi‐square test for inference. Additionally, we will provide the *I* ^2^ statistic as an indicator of the approximate proportion of variation that is due to between‐study heterogeneity rather than a sampling error. Lastly, we will estimate the *τ* ^2^ as an absolute estimation of the magnitude of variation between studies.

#### 3.3.8 Assessment of reporting biases

To estimate the impact from publication bias, we will use funnel plots in combination with trim‐and‐fill analyses. Additionally, we will analyse the risk of publication bias with the Egger regression tests and the Kendall tau test.

#### 3.3.9 Data synthesis

Analyses will include a descriptive summary of the contextual characteristics, methodological characteristics, sample characteristics, and outcome characteristics of the included studies.

PS‐I interventions and control interventions will be compared using averaged effect sizes based on the standardized mean difference, weighted with the inverse of variance method. Separate averages will be reported for each of the three primary outcomes of motivation for learning, conceptual knowledge, and transfer. In turn, for each of these outcomes, separate meta‐analyses will be performed for the comparison of PS‐I interventions with each type of control intervention (As defined in section Why it is important to do this review, four different types of control interventions have been identified: instruction with lecture before problem‐solving, instruction with worked‐examples exploration before problem‐solving, instruction with worked examples exploration before further instruction, and problem‐solving with content guidance before instruction. Yet, other types of control interventions might be identified during the review process).

A random effects model will be assumed. This option was chosen instead of a fixed effects model because we expect that a great variety of factors would influence the effect sizes, and therefore it is difficult to assume a common effect size for the studies (Borenstein, 2010). The 95% confidence intervals will be reported for the averaged effect sizes. Funnel plots will be used to visually represent their aggregation.

The comparison between PS‐I and several types of control activities might be complemented with network meta‐analysis, as long as homogeneity of the comparisons fulfil the transitivity assumption, which will be checked by observing the distribution of significant moderators in each comparison. For the network meta‐analyses, we will report a network plot to describe the direct and indirect evidence available across interventions. Effect sizes between treatments will be reported with 95% confidence intervals using a random effects model, and a *p* ˂ .05 will be considered statistically significant.

Beyond these main analyses, we will conduct exploratory analyses, which will include similar comparisons between PS‐I interventions and control interventions, but we will consider secondary outcomes and studies in which there is not strict equivalence of learning materials between the PS‐I interventions and the control interventions.

#### 3.3.10 Subgroup analysis and investigation of heterogeneity

For all of the separate meta‐analyses in which PS‐I is compared with each of the control activities in each of the three primary outcomes, in cases where we find significant statistical heterogeneity, we will perform moderation analyses to identify factors associated with the efficacy of PS‐I. Correlations between potential moderators will precede these analyses to identify whether the effects of different moderators might be cofounded with each other, and to identify potential groupings of moderating variables.

Moderation analyses will be performed individually for each of the variables discussed in How the intervention might work. Specifically, within design features of PS‐I, we will test for use in the initial problem solving activity of contrasting cases (yes vs. no), metacognitive guidance (yes vs. no), and collaborative work (yes vs. no), and for use in the explicit instruction phase of explanations that build upon students' solutions (yes vs. no). Within contextual factors, we will test for the duration of the intervention in minutes, the average age of the sample in years, and learning domain (math related domain vs. other domains). These individual analyses will also be performed with the general risk of bias variable (low risk vs. some concerns vs. high risk of bias). For the categorical variables we will perform subgroup analyses, and for the continuous variables we will perform individual meta‐regression analyses. Further combinations of moderating variables are not initially hypothesized. A minimum aggregation of three studies will be considered necessary for the analyses to be performed.

#### 3.3.11 Sensitivity analysis

We will conduct sensitivity analyses to determine the impact of several decisions, such as removing studies with outlier effect sizes, removing unpublished studies, removing studies with high risk of bias, or using alternative ways for coding or including moderator variables in the analyses.

#### 3.3.12 Summary of findings and assessment of the certainty of the evidence

This is the protocol for a Campbell review whose objective is exploring the efficacy of the educational strategy of Problem‐solving before Instruction (PS‐I) to promote learning and motivation in child and adult students.

## CONTRIBUTIONS OF AUTHORS

- Content: Catherine Chase, Eduardo González‐Cabañes, Trinidad García, and Jose Cárlos Núñez.
- Systematic review methods: González‐Cabañes, Garcia, Chase, and Núñez.
- Information retrieval: González‐Cabañes, García, and Chase. We count on the advisory assistance of librarians at our universities.

## DECLARATIONS OF INTEREST

None of the researchers involved in the team have financial or personal interests in the results of this review, nor belong to any organization with such interests. All of us have published studies on the problem solving before instruction (PS‐I) method. This review is designed as an independent study and procedures will be detailed to allow replication from perspectives different than ours.

## SOURCES OF SUPPORT

**Internal sources**

- No sources of support provided

**External sources**

- Ministry of Universities of the Government of Spain, Spain
- Scholarship to conduct PhD studies (grant number: FPU16/05802)
- Ministry of Economy, Industry, and Competitiveness of the Government of Spain, Spain
- Research Project (Reference PID2019‐107201GB‐100)
- Principality of Asturias, Spain
- Research Project (Reference: FC‐GRUPIN‐IDI/2018/000199)

## Supporting information

Supporting information.
