# Supplementary material for: PROTOCOL: Problem solving before instruction (PS‐I) to promote learning and motivation in child and adult students
Source: Campbell Syst Rev. 2023 Jul 19;19(3):e1337. doi: 10.1002/cl2.1337 (PMC10357100; doi:10.1002/cl2.1337)
Supplement: Supplementary file 1 — Supporting information. [file CL2-19-e1337-s001.docx]

# Appendices

## 1 PsycINFO Search Strategy

Apa PsycInfo (EBSCOhost, 1806 to June 2022, no restrictions)

| **Search** | **Search Terms** | **Results** |
| --- | --- | --- |
| S14 | S5 AND S10 AND S13 | 634 |
| S13 | S11 OR S12 | 1236190 |
| S12 | DE (“Learning” OR “Academic Achievement” OR “Transfer (Learning)” OR “Comprehension” OR “Concept Formation” OR “Achievement Motivation” OR “Academic Achievement Motivation” OR “Curiosity”) | 206079 |
| S11 | TI (learn* OR knowledge OR transfer* OR motivat* OR curious* OR interest*) OR AB (learn* OR knowledge OR transfer* OR motivat* OR curious* OR interest*) | 1161982 |
| S10 | S6 OR S7 OR S8 OR S9 | 1609 |
| S9 | TI “productive failure” OR AB “productive failure” | 61 |
| S8 | TI (invent* AND prepar* AND learn*) OR AB ( invent* AND prepar* AND learn*) | 275 |
| S7 | TI (Prepar* N3 (problem* OR invent*)) OR AB (prepar* N3 (problem* OR invent*)) | 641 |
| S6 | TI (((problem* OR explor* OR fail* OR impasse OR invent* OR discover*) N3 (before OR after OR prior OR posterior OR later OR earlier)) N3 (instruct* OR worked-example OR teach* OR expla* OR learn*)) OR AB (((problem* OR explor* OR fail* OR impasse OR invent* OR discover*) N3 (before OR after OR prior OR posterior OR later OR earlier)) N3 (instruct* OR worked-example OR teach* OR expla* OR learn*)) | 669 |
| S5 | S1 OR S2 OR S3 OR S4 | 784148 |
| S4 | DE (“Students” OR “College Students” OR “Undergraduate Education” OR “Secondary Education” OR “Primary School Students” OR “Elementary Schools” OR “School Learning” OR “Teaching Methods” OR “Teaching” OR “Classrooms” OR “Problem Solving” OR “Problem Based Learning”) | 293461 |
| S3 | TI ((educat* OR learn*) N2 (institution OR association OR company)) OR AB ((educat*l OR learn*) N2 (institution OR association OR company)) | 10913 |
| S2 | TI ((primary N2 school) OR (elementary N2 school) OR (secondary N2 school) OR (middle N2 school) OR university OR collegue) OR AB ((primary N2 school) OR (elementary N2 school) OR (secondary N2 school) OR (middle N2 school) OR university OR collegue) | 259609 |
| S1 | TI (student* OR pupil*) OR AB (student* OR pupil*) | 574570 |
